# UPR Responsive Genes *Manf* and *Xbp1* in Stroke

**DOI:** 10.3389/fncel.2022.900725

**Published:** 2022-06-15

**Authors:** Helike Lõhelaid, Jenni E. Anttila, Hock-Kean Liew, Kuan-Yin Tseng, Jaakko Teppo, Vassilis Stratoulias, Mikko Airavaara

**Affiliations:** ^1^HiLIFE – Neuroscience Center, University of Helsinki, Helsinki, Finland; ^2^Drug Research Program, Division of Pharmacology and Pharmacotherapy, Faculty of Pharmacy, University of Helsinki, Helsinki, Finland; ^3^Individualized Drug Therapy Research Program, Faculty of Medicine, University of Helsinki, Helsinki, Finland; ^4^Department of Medical Research, Hualien Tzu Chi Hospital, Buddhist Tzu Chi Medical Foundation, Hualien City, Taiwan; ^5^Department of Neurological Surgery, Tri-Service General Hospital, National Defense Medical Center, Taipei, Taiwan; ^6^Drug Research Program, Division of Pharmaceutical Chemistry and Technology, Faculty of Pharmacy, University of Helsinki, Helsinki, Finland

**Keywords:** ARMET, CDNF, ER stress, IRE1, mesencephalic astrocyte-derived neurotrophic factor, unfolded protein response, XBP1

## Abstract

Stroke is a devastating medical condition with no treatment to hasten recovery. Its abrupt nature results in cataclysmic changes in the affected tissues. Resident cells fail to cope with the cellular stress resulting in massive cell death, which cannot be endogenously repaired. A potential strategy to improve stroke outcomes is to boost endogenous pro-survival pathways. The unfolded protein response (UPR), an evolutionarily conserved stress response, provides a promising opportunity to ameliorate the survival of stressed cells. Recent studies from us and others have pointed toward mesencephalic astrocyte-derived neurotrophic factor (MANF) being a UPR responsive gene with an active role in maintaining proteostasis. Its pro-survival effects have been demonstrated in several disease models such as diabetes, neurodegeneration, and stroke. MANF has an ER-signal peptide and an ER-retention signal; it is secreted by ER calcium depletion and exits cells upon cell death. Although its functions remain elusive, conducted experiments suggest that the endogenous MANF in the ER lumen and exogenously administered MANF protein have different mechanisms of action. Here, we will revisit recent and older bodies of literature aiming to delineate the expression profile of MANF. We will focus on its neuroprotective roles in regulating neurogenesis and inflammation upon post-stroke administration. At the same time, we will investigate commonalities and differences with another UPR responsive gene, X-box binding protein 1 (XBP1), which has recently been associated with MANF’s function. This will be the first systematic comparison of these two UPR responsive genes aiming at revealing previously uncovered associations between them. Overall, understanding the mode of action of these UPR responsive genes could provide novel approaches to promote cell survival.

## Introduction: Stroke, MANF, and UPR

Stroke is the second leading cause of death worldwide and the third leading cause of death and disability combined (Collaborators, 2021). Cerebral ischemic stroke is caused by local thrombosis or embolism leading to a lack of blood supply to a focal area of the brain. Globally, 62% of all new strokes are estimated to be ischemic (Collaborators, 2021). Hemorrhagic stroke encompasses the rest of stroke cases, caused by either intracerebral or subarachnoid hemorrhage. Risk factors for stroke include age, arterial atherosclerosis, cardiac diseases, hypertension, diabetes, obesity, smoking, low physical activity, and unhealthy diet. Current treatment practice for ischemic stroke includes reperfusion therapies, which involve the usage of thrombolytic tissue plasminogen activator (tPA) or endovascular thrombectomy (Campbell et al., 2019). However, new treatment strategies are needed, as the recovery from stroke is often incomplete, and there is no drug therapy that could promote the functional recovery of stroke survivors.

Mesencephalic astrocyte-derived neurotrophic factor (MANF) has been originally described as a neurotrophic factor (Petrova et al., 2003) but has subsequently been found to be an important regulator of endoplasmic reticulum (ER) lumen homeostasis (Lindahl et al., 2014) and to have a protective effect on ischemic stroke outcome (Airavaara et al., 2009). The main function of the ER lumen is to maintain protein homeostasis, e.g., folding and quality control of secretory proteins, extracellular matrix proteins, plasma membrane proteins, and ER luminal proteins. There are many excellent reviews on unfolded protein response (UPR) (Hetz et al., 2011, 2020; Walter and Ron, 2011), which is an essential part of maintaining ER lumen and cell homeostasis. The signaling of UPR occurs through three ER transmembrane receptor pathways, inositol-requiring enzyme 1 (IRE1), protein kinase RNA-like ER kinase (PERK), and activating transcription factor 6 (ATF6). Disturbances in ER homeostasis are followed by functional deficits in the cell. If unfolded proteins accumulate in the ER lumen, the IRE1, PERK, and ATF6 pathways signal to the nucleus to induce the transcription-translation of proteins required to handle and/or reduce a load of misfolded proteins to overcome the stressful event, leading to cell survival. In addition, the modulation of ER-homeostasis *via* the IRE1/X-box binding protein 1 (XBP1) pathway leads to immune regulation (Hetz et al., 2011; Pramanik et al., 2018). In case of continuous and chronic ER stress, cell can undergo necroptosis or mitochondrial apoptosis (Iurlaro and Munoz-Pinedo, 2016). UPR can be activated by different mechanisms interfering with protein folding homeostasis such as changes in ER luminal Ca^2+^ concentration, energy or glucose depletion, lipid accumulation, and viruses (Foufelle and Fromenty, 2016). UPR can be pharmacologically induced, e.g., by thapsigargin, which depletes ER Ca^2+^, or tunicamycin, which inhibits N-glycosylation of newly synthetized proteins, leading to accumulation of unfolded proteins. However, the physiological relevance of these pharmacological manipulations needs to be considered with caution. Moreover, the quantity of protein secretion is likely the critical factor in how detrimental chronic UPR is. It should be noted that in neurons, protein secretion quantity is rather small, but quality demand together with the structural complexity of cells can lead to detrimental effects of chronic UPR (Sree et al., 2021). An essential player in UPR regulation is GRP78/BiP, an ER resident chaperone importing nascent proteins from cytosol to the ER, which under normal conditions also binds to UPR receptors (Bertolotti et al., 2000; Kopp et al., 2019). GRP78 binding to unfolded proteins is ADP/ATP-dependent, and the activity of GRP78 as a chaperone is controlled by different cofactors (e.g., GRP170 (alias HYOU1) and ORP150) and SIL1 (Behnke et al., 2015). Interestingly, there is evidence that signaling *via* the IRE1/XBP1 and ATF6 pathways is protective in ischemic injury (Glembotski et al., 2019).

The primary focus of this review is to highlight the importance of the UPR and IRE1/XBP1 pathway in brain injuries and the potential of MANF as a therapeutic agent in stroke. We will focus on comparing the role of XBP1 and MANF in stroke and the function of MANF as a regulator of inflammation and neurogenesis.

## Mesencephalic Astrocyte-Derived Neurotrophic Factor

MANF and cerebral dopamine neurotrophic factor (CDNF) are proteins with pleiotropic effects on various disease models (Lindahl et al., 2017; Albert and Airavaara, 2019; Jäntti and Harvey, 2020; Lindholm and Saarma, 2021) and form together a family of proteins (amino acid identity is 59%) (Lindholm et al., 2007). MANF was originally discovered and named ARMET (arginine-rich, mutated in early-stage tumor) (Shridhar et al., 1996) but was subsequently renamed MANF when the protein was first successfully isolated from a mesencephalic astrocyte cell line, and its neurotrophic properties were reported using dopaminergic neuronal cultures (Petrova et al., 2003). Since the initial *in vitro* studies that suggested selectivity for protecting dopaminergic neurons (Petrova et al., 2003), follow up studies have found very little activity when the MANF protein is applied into cell culture media (Hellman et al., 2011). Because MANF lacks the plasma membrane receptor – however, notice some binding to neuroplastin (Yagi et al., 2020) – one can argue how accurate the name neurotrophic factor is. Furthermore, MANF’s very high mRNA expression levels in adult mouse brain and in various other secretory tissues suggest that it is not acting as a neurotrophic factor and definitely not as a growth factor (Zhang et al., 2014; Danilova et al., 2019b; Karlsson et al., 2021). Moreover, the part in MANF’s name referring to mesencephalic astrocytes is not that accurate either, since *Manf* mRNA is expressed at very high levels in all cells (Lindholm et al., 2008; Zhang et al., 2014; Danilova et al., 2019b; Karlsson et al., 2021).

**FIGURE 1:**
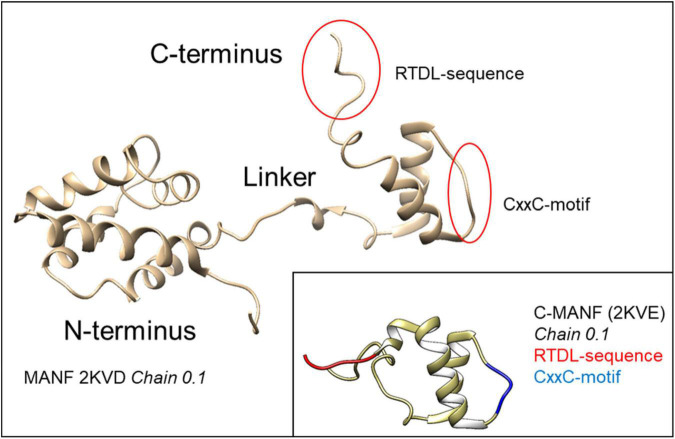
Structure-function relationship of mesencephalic astrocyte-derived neurotrophic factor (MANF). The figure is based on the NMR structures of MANF and C-MANF (Hellman et al., 2011) (PDB codes: 2KVD and 2KVE, respectively. Image created with Chimera 1.16). The C-terminal RTDL-sequence of MANF corresponds to the canonical ER-retrieval signal “KDEL”.

MANF is a small soluble protein (18–20 kDa) with a signal peptide targeting the protein into the ER lumen (Petrova et al., 2003; Mizobuchi et al., 2007). The MANF protein is comprised of two domains: N-terminal Saposin-like (Parkash et al., 2009) and the C-terminal SAP-like domain connected by a flexible linker region (Hellman et al., 2011; Figure 1). Other proteins with Saposin-like domain bind lipids and, indeed, the N-terminal domain of MANF have been shown to bind sulfatides (3-O-sulfogalactosylceramide) (Bai et al., 2018) that are enriched in myelin (Grassi et al., 2015). The structure of the C-terminal domain of MANF obtained by NMR was strikingly similar to the Ku70 C-terminal SAP-like domain. Ku70C and other similarly structured proteins bind DNA and proteins (Hellman et al., 2011); however, the Ku70-related anti-apoptotic effect of MANF has not been detected. MANF has 8 cysteines and contains two CxxC-motifs, one per domain. Proteins with a CxxC-motif are abundant in the ER, e.g., reductases and protein disulfide isomerases (PDIs) that can help to fold proteins (Kersteen and Raines, 2003). Thus, the presence of an CxxC-motif in MANF could indicate a reductase or disulfide isomerase activity. This hypothesis has been tested several times with no evidence of PDI activity in MANF. Importantly, mutation of the C-terminal domain CxxC-motif (Figure 1) erases MANF’s protective functions regardless of delivery method: the neuroprotective activity induced by gene therapy *in vitro* and with recombinant protein *in vivo* (Matlik et al., 2015). It should be noted that MANF’s protective activity by gene therapy *in vitro* was abolished with the deletion of the C-terminal RTDL domain, but the protein was biologically active and showed protective effects on ischemic stroke *in vivo*. Thus, only the C-terminal CxxC is essential for the neuroprotective activity of MANF in ischemic stroke *in vivo* (Matlik et al., 2015) and antagonizes cell death (Božok et al., 2018).

Interestingly, MANF is an UPR activated gene (Tseng et al., 2017; Hartman et al., 2019; Jäntti and Harvey, 2020; Pakarinen et al., 2020, 2022). MANF works under reductive ER stress in the heart as well (Arrieta et al., 2020). Previous studies have shown that MANF interacts with ER-resident proteins: GRP78 (Glembotski et al., 2012), PDI6 (Bell et al., 2018) and CH60, KCRB, and PGAM (Eesmaa et al., 2021). The C-terminal domain of MANF was proposed to bind the N-terminal nucleotide-binding domain of GRP78 and to prolong the interaction of GRP78 with its “client” proteins by nucleotide exchange of GRP78 (Yan et al., 2019), indicating that the biological function of MANF in the ER lumen is to act as an ER lumen chaperone and to maintain ER homeostasis. However, MANF’s interaction with GRP78 is not required for neuroprotective activity *in vitro* by gene therapy when microinjected as plasmid to the nucleus of the neuron (Eesmaa et al., 2021). How these results would relate to effects of the recombinant MANF protein without the ER signal sequence and the possibility to enter the ER lumen in animal models *in vivo* remains unclear and requires further studies.

**FIGURE 2:**
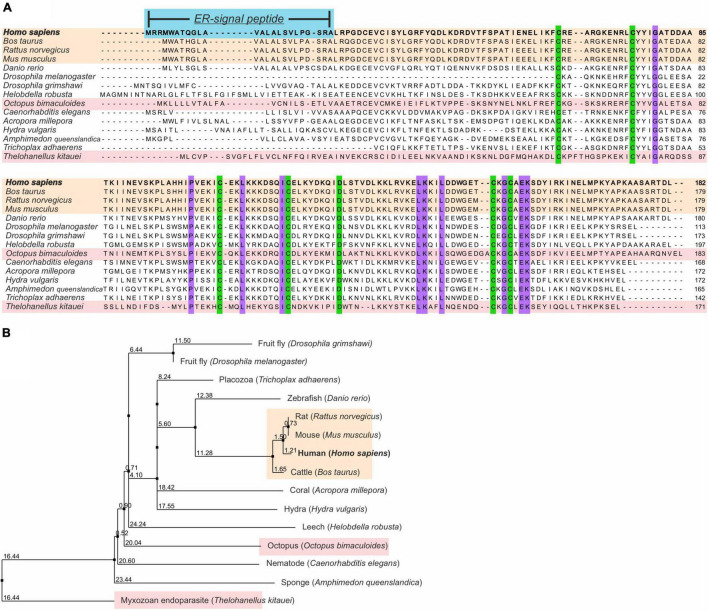
Phylogenetic analysis of MANF. **(A)** Vertebrate MANF orthologs (cream boxes) were obtained by NCBI, as calculated by NCBI’s Eukaryotic Genome Annotation pipeline for the NCBI Gene dataset. Non-vertebrate MANF orthologs were obtained with the NCBI “similar gene” pipeline. For two of the metazoans presented (pink boxes), human protein sequence was blasted in the EnsemblMetazoan database (metazoa.ensembl.org). Protein sequences were aligned using ClustaOWS in Jalview v. 2.10.5 (Clamp et al., 2004). Conserved amino acids in all organisms are highlighted in purple or green for cysteines. Numbers denote the amino acid number. **(B)** Phylogenetic tree of MANF is based on protein sequence similarity. It was calculated from distance matrices determined from % identity using the neighbor joining algorithm. Each number is a score, and each branch is an additive allowing for comparison of distances in the tree branches. Used sequence IDs: *Homo sapiens* (T1FAB3), *Bos Taurus* (Q9N3B0), *Rattus norvegicus* (A0A0C2MNP5), *Mus musculus* (A0A0L8FVI8), *Danio rerio* (B3RIB4), *Drosophila grimshawi* (B4JT39), *Drosophila melanogaster* (Q9XZ63), *Helobdella robusta* (Q3TMX5), *Octopus bimaculoides* (P55145), *Caenorhabditis elegans* (P80513), *Acropora millepora* (F1QDQ5), *Hydra vulgaris* (B2RZ09), *Amphimedon queenslandica* (T2MFG7), *Trichoplax adhaerens* (A0A1 × 7TYG8), and *Thelohanellus kitauei* (LOC114953444).

MANF is an evolutionarily highly conserved protein found in many branches of life, from humans to sponges. The amino acid similarity of MANF between human and sponges is ∼50–59%, while the similarity to leech and octopus is ∼41 and 45%, respectively (Figure 2). Homology is selectively maintained because of structural or functional constraints, e.g., cysteines present in MANF are important for building disulfide bonds, which in turn is important for the function of the protein.

MANF expression is induced by ATF6 and XBP1 activation (Lee et al., 2003; Shaffer et al., 2004; Tadimalla et al., 2008). *Manf* expression was shown to be upregulated most efficiently by the transcription factor ATF6α and moderately by ATF6β and spliced XBP1 (XPB1s) in Neuro2a cells (Oh-Hashi et al., 2013). The *Manf* promoter sequence contains ERSE binding elements, and *Manf* was suggested to be induced upon ER stress *via* ER stress response element II (ERSE-II) (Mizobuchi et al., 2007). In a later study, XBP1s was found to induce MANF by binding ERSE-I in the *Manf* promoter region (Wang et al., 2018). MANF is involved in protein folding homeostasis in the ER and is able to restrict ER stress *in vivo* (Lindahl et al., 2014; Hartman et al., 2019). Still, the molecular mechanism of how the recombinant MANF protein facilitates neuroprotective effects *in vivo* remains unknown. However, immunomodulation may also play a role in mediating MANF’s cytoprotective effects.

**FIGURE 3:**
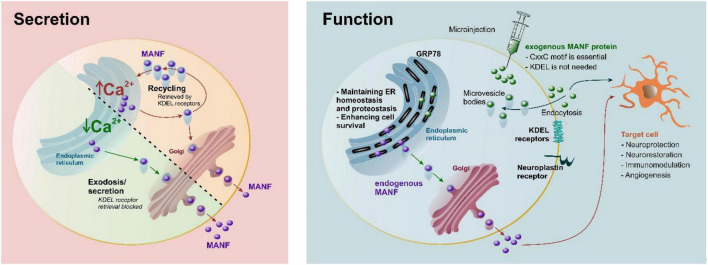
Secretion and proposed function of MANF. Under normal conditions, MANF is maintained in the endoplasmic reticulum (ER) by KDEL receptor-mediated retrieval and secreted in response to Ca^2+^ depletion. MANF maintains ER homeostasis and proteostasis, as well as enhances cell survival. A CxxC-motif is essential for neuroprotection for both endogenous and exogenous MANF, while the C-terminal RTDL sequence of MANF (corresponding to the canonical KDEL ER-retrieval signal) is not needed for the neuroprotective effect of exogenous MANF (Matlik et al., 2015). It is possible that KDEL and neuroplastin receptors may participate in internalization of extracellular MANF, and interaction with sulfatides may be important for internalization. It should be emphasized the majority of KDEL receptors are in the Golgi, and only a fraction of them can be at the plasma membrane at any given time point. Purple circles, endogenous MANF; green circles, recombinant exogenous MANF.

Under normal conditions, a major part of expressed MANF has been shown to localize in the ER by co-staining with ER-resident proteins Hrd1, PDI, and GRP78 (Mizobuchi et al., 2007; Apostolou et al., 2008; Tadimalla et al., 2008; Matlik et al., 2015) and only a small amount is secreted (Apostolou et al., 2008; Tadimalla et al., 2008; Henderson et al., 2013; Figure 3). However, MANF secretion is significantly enhanced upon ER Ca^2+^ depletion but not upon protein misfolding or alteration of ER redox status (Glembotski et al., 2012; Henderson et al., 2013), indicating ER stress *per se* is not a trigger for MANF secretion but ER Ca^2+^ depletion is needed (Figure 3). Extracellular levels of MANF also increase in response to cell death. In addition, the action and secretion of MANF could vary in different cells, as MANF (and CDNF) regulates ER homeostasis in a tissue-specific manner (Pakarinen et al., 2022).

While the exact mechanism for extracellular MANF is not fully resolved, it has become clear that ER-luminal MANF has an active role in maintaining ER lumen proteostasis (Figure 3). Therefore, it is not surprising that MANF is expressed widely in different tissues and is particularly highly expressed in metabolic and secretory tissues, such as those of the liver and pancreas, and in tissues related to the immune system, such as those of the bone marrow, and lymphoid tissues (Uhlen et al., 2015; Danilova et al., 2019b; Karlsson et al., 2021). The MANF protein is also found in circulating blood (Galli et al., 2016, 2019a,b; Wei et al., 2020; Wang et al., 2021c). In the brain, the MANF protein is expressed mainly in neurons under normal conditions (Lindholm et al., 2008; Tseng et al., 2018; Danilova et al., 2019b) but *Manf* mRNA is also expressed at high levels in astrocytes, microglia, oligodendrocytes, and endothelial cells (Zhang et al., 2014; Karlsson et al., 2021). MANF has been shown to protect against many types of cerebral injuries in *in vivo* disease models, including ischemic stroke (Airavaara et al., 2009, 2010; Yang et al., 2014; Wang et al., 2016; Matlik et al., 2018), hemorrhagic stroke (Xu et al., 2018; Li et al., 2019), traumatic brain injury (Li et al., 2018), and Parkinson’s disease (Voutilainen et al., 2009; Hao et al., 2017). However, MANF has cytoprotective properties not only in the brain but also in the heart (Glembotski et al., 2012), retina (Neves et al., 2016; Gao et al., 2017; Lu et al., 2018), pancreas (Lindahl et al., 2014; Danilova et al., 2019a; Montaser et al., 2021), liver (Sousa-Victor et al., 2019), and kidney cells (Park et al., 2019).

Cerebral MANF expression has been shown to occur already during early embryonic development: mRNA at embryonic day (E) 7.5 and protein at E9.5 in mice (Danilova et al., 2019b). A study on postnatal cerebral MANF expression in rats revealed that MANF protein expression is highest during the early postnatal development on days P3–5 and is significantly decreased when adulthood is reached (Wang et al., 2014). Notably, the expression of the MANF protein has been shown to further decrease with aging. A recent study found significantly reduced MANF protein levels in the brain of 18- to 20-month-old mice compared to 2- to 3-month-old mice (Han et al., 2021) and additional groups of 6- and 9-month-old mice (Liu et al., 2021). MANF protein expression has been also shown to decrease with aging in the fruit fly, in mouse plasma, retinal choroid, white adipose tissue, skin, liver, and muscle tissues, and in human serum and retinal choroid (Sousa-Victor et al., 2019; Neves et al., 2020; Liu et al., 2021). Decreased MANF expression could predispose for many age-related diseases and worsen the outcome of stroke.

MANF is a highly soluble protein (Mizobuchi et al., 2007) and spreads well in the brain parenchyma after intracerebral recombinant protein injection (Voutilainen et al., 2009, 2011). This high volume of distribution and spreading in the brain parenchyma is also evident with CDNF (Mätlik et al., 2017). Compared to glial cell line-derived neurotrophic factor (GDNF), the MANF (and CDNF) protein has significantly better tissue diffusion properties (Voutilainen et al., 2009, 2011; Mätlik et al., 2017).

How the exogenous MANF protein affects/enters cells is not fully understood. MANF is suggested to bind neuroplastin in the plasma membrane (Yagi et al., 2020); however, the binding efficacy is far from that of classical plasma membrane receptors. Furthermore, it should be kept in mind that in previous studies the radiolabeled MANF failed to bind anything (Hellman et al., 2011). Endogenous MANF is retrieved back to the ER lumen *via* KDEL receptors (KDELR) because of its C-terminal RTDL motif. MANF may also be internalized into a cell *via* KDEL-receptors, as they can be found in the plasma membrane fraction, but KDELRs are not classically thought to be transmembrane receptors in the plasma membrane (Henderson et al., 2013). Indeed, after ER stress and ER Ca^2+^ depletion, the level of KDELRs present in the plasma membrane may be increased. Due to the fusion of secretion vesicles in the plasma membrane, this seems quite likely. However, MANF without the RTDL sequence is still protective *in vivo* in ischemic stroke (Matlik et al., 2015), indicating that KDELRs are not necessary for mediating MANF’s neuroprotective effects *in vivo*. Also, the fractalkine receptor CX3CR1 expressed by immune cells has been implicated as a mediator of MANF’s cytoprotective effects on mouse retina (Neves et al., 2016). In addition, sulfatides have been shown to bind the extracellular MANF protein and to mediate its endocytosis into cells and were, thus, suggested to function as cell surface receptors (Bai et al., 2018). Furthermore, the cytoprotective effect of extracellular MANF was sulfatide-dependent, and the sulfatide-bound MANF was able to reduce ER stress in both *C. elegans* and in mammalian cells. However, it is still not known how MANF would enter the cell after binding to sulfatides. Interestingly, the recombinant CDNF protein, when delivered into the brain parenchyma, enters neurons very efficiently *via* unspecific endocytosis, and most CDNF was found in multivesicular bodies (Mätlik et al., 2017). Likely, a similar phenomenon also occurs with MANF (Figure 3); but so far, there is no evidence that neither protein would end up in the ER lumen after endocytosis (Mätlik et al., 2017), and how the transport would be possible without the ER signal peptide is not known. A recent study indicates that the recombinant MANF protein, when added to cell culture media, was protective only on stressed dopamine neurons treated with thapsigargin, a SERCA pump inhibitor (Eesmaa et al., 2021). In the future, studies should be conducted with radiolabeled MANF to determine the affinity together with competitive binding and deletion of KDELRs and/or neuroplastin. In summary, there has been tremendous progress in determining the actions of ER luminal MANF during the past 10 years. However, it is rather likely that the pleiotropic protective effects of the extracellular recombinant MANF protein (without the ER signal peptide) are mediated differently, and more studies are needed to discover the mechanism of MANF’s cellular uptake and possible cell surface receptors.

## Summary of MANF Neuroprotection Studies

MANF is an ER stress-inducible protein (Apostolou et al., 2008) that conveys neuroprotective and neurorestorative properties. Microinjection of recombinant MANF or MANF overexpression by plasmid microinjection into the nucleus inhibits apoptosis in primary neuronal cultures treated with the UPR inducers thapsigargin and tunicamycin, topoisomerase II inhibitor etoposide, and protein kinase inhibitor staurosporine, or nerve growth factor (NGF) deprivation in culture media (Hellman et al., 2011; Matlik et al., 2015; Eesmaa et al., 2021). Extracellularly applied MANF was shown to reduce apoptosis and downregulate the ATF6 and IRE1 UPR pathways in thapsigargin-treated dopaminergic primary cultures (Eesmaa et al., 2021). The protective effect of both intracellularly and extracellularly applied MANF in primary neuronal cells was inhibited by IRE1 and PERK inhibitors (Eesmaa et al., 2021), indicating that MANF’s cytoprotective effect is mediated by UPR regulation. However, in 6-OHDA treated SH-SY5Y cells, UPR downregulation was observed only after MANF overexpression and not after applying the recombinant protein extracellularly into media (Hao et al., 2017). In the *in vivo* 6-OHDA toxin model of Parkinson’s disease, the recombinant MANF protects the dopaminergic cell bodies of the substantia nigra (Voutilainen et al., 2009; Hao et al., 2017). MANF not only prevents the degeneration of dopaminergic neurons but also potentiates stimulus-evoked dopaminergic neurotransmission, thereby enhancing dopamine turnover in the brain of freely moving rats without lesion (Renko et al., 2018). Also, *in vitro*, recombinant MANF protects the SH-SY5Y neuroblastoma cell line from 6-OHDA-induced apoptosis (Huang et al., 2016; Hao et al., 2017; Sun et al., 2017; Zhang et al., 2017). In *D. melanogaster*, the *Manf* homolog *DmManf* is required for the maturation and maintenance of dopaminergic neurites during the embryonic stage, implying the importance of MANF in Parkinson’s disease, where dopaminergic neuronal loss is the major factor (Palgi et al., 2009). Furthermore, neuronal MANF knockdown sporadically affects dopaminergic neuron development, suggesting a cell autonomous function in these cells (Stratoulias and Heino, 2015a). Similarly in zebrafish, embryonic *Manf* knockdown results in dopaminergic neuron impairment, a phenotype which is rescued by exogenous *Manf* mRNA (Chen et al., 2012). In *C. elegans*, mutation of the *Manf* homolog *manf-1* showed increased α-synuclein accumulation in the body wall muscle cells together with increased ER stress (Richman et al., 2018). Inability to cope with the UPR machinery might, in turn, lead to degeneration of dopaminergic neurons, as dopaminergic degeneration was found to occur with age in the *manf-1* mutant *C. elegans.* Also, deletion of MANF in *C. elegans* abrogated tunicamycin-induced toxicity, and even high concentrations did not decrease survival (Hartman et al., 2019). MANF overexpression or the recombinant MANF protein has been shown to protect against Aβ toxicity in N2a cells and SH-SY5Y cells possibly by inhibiting Aβ-induced ER stress, whereas knockdown of the *Manf* gene was shown to aggravate Aβ accumulation and ER stress (Xu et al., 2019). Additionally, recombinant MANF has been shown to be neuroprotective in a rat model of traumatic brain injury, where the protective effect was attributed to increased BBB integrity and decreased activation of the NF-κβ signaling pathway (Li et al., 2018). MANF was speculated to inhibit the apoptosis of endothelial cells constituting the BBB by downregulating ER stress and/or inflammation. MANF has also shown protective effects on a mouse model of multiple sclerosis, where recombinant MANF-treated mice showed less motor deficits compared to vehicle-treated mice in an early time-point after experimental autoimmune encephalomyelitis induction (Nam et al., 2021).

## MANF and XBP1 in Stroke

### MANF

MANF expression is upregulated by ischemia (Table 1). A short 10-min global forebrain ischemia in rat transiently increased neuronal *Manf* mRNA levels at 24 h in the hippocampus and the levels returned to baseline by 1-week post-ischemia (Lindholm et al., 2008). The protein expression of neuronal MANF was found elevated 2–48 h post-stroke after transient middle cerebral artery occlusion (MCAo) in rats, and most prominently in the peri-infarct region (Apostolou et al., 2008; Yu et al., 2010). Interestingly, Shen et al. reported that the MANF protein was upregulated not only in neurons of the ischemic cortex but also in CD68-positive microglia/macrophages and in oligodendrocytes 24 h after transient MCAo in rat (Shen et al., 2012). MANF expression colocalized with GRP78 in CD68-positive cells, indicating UPR activation in microglia/macrophages after ischemic stroke. Minor MANF expression was also found in astrocytes of the ischemic cortex but not in the normal healthy brain (Shen et al., 2012). In a more recent study, Belayev et al. found that docosahexaenoic acid increased MANF protein expression in neurons and astrocytes of the peri-ischemic region 24 h after transient MCAo in rats (Belayev et al., 2020). Yang et al. showed increased MANF expression in microglia/macrophages at the same time point after transient MCAo, and a further increase in MANF positive microglia/macrophages was seen when animals with stroke were pretreated with bone marrow mesenchymal stem cells (Yang et al., 2020). When we overexpressed the MANF transgene in the rat cortex before cortical stroke induction by distal MCAo (dMCAo), the expression pattern of transduced MANF was changed 6 h after ischemia, causing redistribution of MANF from the cell soma to the processes of neurons and glia (Airavaara et al., 2010). Furthermore, we observed a punctate pattern of transduced MANF after ischemia, suggestive that the MANF protein is released from cells after ischemia or locally translated in axonal/dendritic processes. The observed phenomena may be related to “ER exodosis,” the exit of ER luminal proteins upon ER stress (Henderson et al., 2021), as MANF is known to be secreted from cells upon ER Ca^2+^ depletion (Glembotski et al., 2012; Henderson et al., 2013).

**TABLE 1:** Effect of MANF in stroke.

Model	Treatment	Effect	References
***In vivo Studies***			
1 h MCAo in aged mice	rhMANF 2 h post-stroke	Functional recovery ↑ Infarct volume ↓ IL-6, IL-1β, TNF-α↓, BBB integrity ↑	Han et al., 2021
2 h MCAo in rat	DHA *i.v.* 3 h post-stroke	MANF in neurons and astrocytes ↑ Neurobehavioral recovery ↑ TREM2 in microglia ↓ Infarct size ↓	Belayev et al., 2020
Permanent MCAo in rat	rhMANF 24 h post-stroke	Neurobehavioral recovery ↑ CD34 ↑ Regional cerebral blood flow ↑ Vessel surface area ↑ Microvessel branch points ↑ VEGF pathway activation	Gao et al., 2020
1 h dMCAo in rat	AAV1-MANF 2 days post-stroke	Some immune response-related transcripts ↑ S100A8; S100A9 ↓	Teppo et al., 2020
2 h MCAo in rat	MANF-knockdown BMSC transplant 1-day pre-stroke	M1 markers ↑ M2 markers ↓ BMSC-induced functional recovery ↓ MANF-mediated MANF upregulation ↓	Yang et al., 2020
1.5 h dMCAo in rat	rhMANF 7 days post-stroke	Functional recovery ↑	Anttila et al., 2019
Subarachnoid hemorrhage in rat	rhMANF at stroke	p-Akt, p-MDM2, Bcl-2 ↑ BBB integrity ↑ P53, Bax, CC3, MMP9 ↓ Neurological deficit ↓ Effects reversed by MK2206	Li et al., 2019
1 h dMCAo in rat	AAV7-MANF 2 days post-stroke	Functional recovery ↑ Phagocytic macrophages ↑ *Complement 3* and *Emr1* ↑	Matlik et al., 2018
1.5 h dMCAo in rat	chronic rhMANF 3–16 days post-stroke	Functional recovery ↑	Matlik et al., 2018
Permanent dMCAo in mouse	*Nestin^Cre/+^*:Manf *^fl/fl^* MANF KO	Infarction volume ↑ *Manf* mRNA ↑ in *Manf ^fl/fl^* control mouse cortex	Matlik et al., 2018
1.5 h dMCAo in rat	rhMANF 3-, 7-, and 10-days post-stroke or continuously on days 3–16 post-stroke	Migration of DCX^+^ cells toward corpus callosum and infarct boundary ↑	Tseng et al., 2018
Intracerebral hemorrhage in rat	rhMANF 1-day post-stroke	p-Akt, p-MDM2, Bcl/Bax ratio ↑ Neurological function ↑ P53, caspase-3, neuronal death ↓ Effects reversed by MK2206	Xu et al., 2018
1.5 h MCAo in rat	rhMANF 3 h post-stroke	Neurological function ↑ Mortality ↓ Infarct volume and brain tissue injury ↓	Wang et al., 2016
1 h dMCAo in rat	rhMANF C-terminal mutations 20 min pre-stroke	CKGC sequence required for neuroprotective activity, RTDL not	Matlik et al., 2015
2 h MCAo in rat	rhMANF 20 min pre-stroke	Neuronal loss ↓ Caspase-3 cleavage, apoptosis ↓ BIP/GRP78, p-IRE1, and XBP1 ↓	Yang et al., 2014
2 h or 4 h MCAo in rat		MANF is primarily expressed in neurons MANF expression in neurons, microglia/macrophages, and oligodendrocytes. Only mild MANF upregulation in astrocytes	Shen et al., 2012
1 h dMCAo in rat	AAV7-MANF 1 week pre-stroke	Behavioral recovery ↑ Infarction volume ↓ MCAo causes redistribution of MANF immunoreactivity after overexpression	Airavaara et al., 2010
2 h or 4 h MCAo in rat		MANF expression in neurons and glial cells ↑ 2–48 h post-MCAo	Yu et al., 2010
***In vivo studies***			
1 h dMCAo in rat	rhMANF 20 min pre-stroke	Behavioral recovery ↑ Infarction volume ↓ Apoptosis ↓	Airavaara et al., 2009
10 min oCCA *(Global forebrain ischemia)* in rat		MANF mRNA ↑ at 24 h, expression mostly neuronal	Lindholm et al., 2008
***In vitro studies***			
Thapsigargin or OGD on SH-SY5Y cell culture	Various compounds for 16 h pretreatment	Compounds that stabilize the ER-resident proteome reverse MANF secretion	Henderson et al., 2021
OGD on senescent bEnd.3 cells	rhMANF 24 h pretreatment	IL-6, IL-1β, TNF-α↓ TLR4, MyD88, nuclear NF-κB p65 ↓ ZO-1, occludin ↑	Han et al., 2021
LPS-stimulated primary microglia or HAPI cell culture	Microglia: BMSC-co-culture 1-day post-LPS; HAPI: PDGF-AA 1-day post-LPS	Microglia: MANF ↑, reversed if BMSCs are MANF-knockdown HAPI: MANF ↑, miR-30a ↓	Yang et al., 2020
OGD on NSC culture, SVZ explants	NSC: rhMANF 15 min pre-OGD; SVZ: LV-hMANF	NSC: neuronal and glial differentiation ↑, STAT3 activation SVZ: Cell migration ↑, STAT3 and ERK1/2 activation	Tseng et al., 2018
Etoposide or thapsigargin on sympathetic or sensory neuron culture	Plasmid DNA of MANF C-terminal mutants at treatment	CKGC sequence required for neuroprotective activity RTDL sequence required for ER retention and neuroprotection in sympathetic but not sensory neurons	Matlik et al., 2015
OGD in primary rat astrocytes	rhMANF	IL-1β, IL-6, TNF-α↓ GRP78, NF-κB p65 ↓	Zhao et al., 2013
Primary glial cell culture	Starvation, MG132, or tunicamycin	MANF expression ↑	Shen et al., 2012
Hypoxia on primary cortical neuron culture	AAV7-MANF pre-hypoxia	Hypoxia causes redistribution of MANF immunoreactivity after overexpression	Airavaara et al., 2010
Serum deprivation or tunicamycin on SH-SY5Y culture, tunicamycin on primary neuron culture	rhMANF for 2 weeks pretreatment	MANF expression after tunicamycin ↑ rhMANF: neuronal survival ↑	Yu et al., 2010

*AAV, adeno-associated virus; Bax, Bcl-2-associated X protein; BBB, blood-brain barrier; Bcl-2, B-cell lymphoma 2; BIP/GRP78, binding immunoglobulin protein/glucose-regulated protein 78; BMSC, bone marrow mesenchymal stem cell; CC3, cleaved caspase 3; oCCA, occlusion of common carotid artery; DCX^+^, double cortin-positive neurons; DHA, docosahexaenoic acid; dMCAo, distal middle cerebral artery occlusion; Emr1, EGF-like module-containing mucin-like hormone receptor-like 1; ER, endoplasmic reticulum; ERK1/2, Extracellular signal-regulated protein kinase 1/2; HAPI, highly aggressively proliferating immortalized rat microglia; IL, interleukin; i.v., intravenous; KO, knockout; LPS, lipopolysaccharide; LV, lentivirus; M1, classically activated microglia/macrophages; M2, alternatively activated microglia/macrophages; MANF, mesencephalic astrocyte-derived neurotrophic factor; MCAo, middle cerebral artery occlusion; MMP9, matrix metallopeptidase 9; MyD88, myeloid differentiation factor 88; NF-κB, nuclear factor kappa-light-chain-enhancer of activated B cells; NSC, neural stem cell; OGD, oxygen-glucose deprivation; P53, tumor suppressor p53; p-Akt, phosphorylated protein kinase B; PDGF-AA, platelet-derived growth factor-AA; p-IRE1, phosphorylated IRE1; p-MDM2, phosphorylated murine double minute 2; rhMANF, recombinant human MANF; S100A8/9, calgranulin A/B; STAT3, signal transducer and activator of transcription 3; SVZ, subventricular zone; TLR4, toll-like receptor 4; TREM2, triggering receptor expressed on myeloid cell 2; VEGF, vascular endothelial growth factor; XBP1, X-box binding protein; ZO-1, zonula occludens-1; TNF-a, tumor necrosis factor a.*

Like cerebral ischemia, myocardial ischemia was shown to upregulate MANF protein levels (Tadimalla et al., 2008). Increased MANF protein levels were found in the peri-infarct area 4–14 days after permanent myocardial infarction in mice (Tadimalla et al., 2008). Additionally, hypoxia in rat primary retinal ganglion cells increased *Manf* mRNA and protein levels 24–48 h after hypoxia (Gao et al., 2016). Furthermore, hemorrhagic stroke has been shown to increase MANF protein expression, mainly in neurons (Xu et al., 2018; Li et al., 2019). In both intracerebral hemorrhage and subarachnoid hemorrhage rat models, MANF protein expression increased 3 h after hemorrhage, peaked at 24 h, and remained elevated up to 72 h post-hemorrhage in the peri-hematoma area.

Collectively, stroke robustly increases the expression of MANF in animal models. Many studies have focused on analyzing neuronal MANF expression, but prominent upregulation in glia has also been reported. However, studies on post-ischemic MANF expression have focused on acute time-points, and a thorough long-term study on MANF expression after ischemic stroke in animal models and in ischemic stroke post-mortem patient samples is further warranted.

When exogenous MANF is administered into the brain *via* a viral vector or as a recombinant protein before or a few hours after stroke, it has neuroprotective effects on ischemic stroke models (Airavaara et al., 2009, 2010; Yang et al., 2014; Matlik et al., 2015; Wang et al., 2016; Han et al., 2021). Neuroprotection can be observed both at the tissue level as reduced infarction volume and at the behavioral level as improved functional recovery. MANF can inhibit apoptosis/necrosis in the ischemic brain (Airavaara et al., 2009; Yang et al., 2014), and the CxxC-motif is indispensable for the neuroprotective effect of MANF in ischemic stroke, possibly because of its importance in maintaining MANF’s structural conformation (Matlik et al., 2015). However, in the rat dMCAo model, the RTDL sequence is not needed for neuroprotection, implying that KDEL receptors do not have a significant role in mediating MANF’s neuroprotective effect on cerebral ischemia (Matlik et al., 2015). Taking a different approach, Belayev et al. used docosahexaenoic acid (DHA) to increase endogenous MANF protein expression after transient MCAo and observed neuroprotection and enhanced neurogenesis associated with DHA therapy (Belayev et al., 2020). An important study on 18- to 20-month-old mice was conducted recently by Han et al. and showed that the recombinant MANF protein given 2 h post-MCAo is also neuroprotective in the aging brain (Han et al., 2021). MANF treatment also increased the BBB integrity of aged mice. Moreover, endogenous neuronal *Manf* is protective against ischemic stroke, as we have shown that *Nestin^Cre/+^*:Manf*^flox/flox(fl/fl)^* mice with conditional deletion of *Manf* from the neural lineage cells have larger infarcts than *Manf^fl/fl^* control mice (Matlik et al., 2018). In *Manf* knockout (KO) mouse primary neuronal stem cell culture, extracellularly applied recombinant MANF protein protected the KO cells from apoptosis after oxygen-glucose deprivation (Tseng et al., 2018). The cytoprotective effect of MANF against ischemia is not limited to neuronal cells, but the recombinant MANF protein reduced the myocardial infarct size in a mouse model of transient myocardial ischemia (Glembotski et al., 2012) as well as reduced hypoxia-induced apoptosis in rat retinal ganglion cells *in vivo* and *in vitro* (Gao et al., 2017). Furthermore, MANF is neuroprotective in hemorrhagic stroke (Xu et al., 2018; Li et al., 2019). Intracerebroventricular injection of the recombinant MANF protein 1 h after intracerebral hemorrhage induction in rats improved neurological function, decreased apoptosis, and activated Akt in the peri-hematoma area (Xu et al., 2018). Comparable results were found in a rat model of subarachnoid hemorrhage, and an additional finding of MMP-9 downregulation suggested MANF may protect the integrity of the BBB (Li et al., 2019).

Notably, post-stroke MANF administration promotes functional recovery (Matlik et al., 2018; Anttila et al., 2019). We overexpressed the MANF protein in the peri-infarct area 2 days after dMCAo by intracerebral AAV7-MANF injection and found improved neurological function already on day 4 and up to day 14 post-stroke (Matlik et al., 2018). Similarly, chronic infusion of the recombinant MANF protein into the peri-infarct area starting 3 days post-dMCAo and continuing for 14 days promoted neurological recovery (Matlik et al., 2018). Even a single intracranial MANF recombinant protein injection 7 days post-stroke was able to alleviate neurological deficits a week later (Anttila et al., 2019).

The mechanisms by which MANF promotes recovery after the acute phase of stroke remain poorly understood. MANF represents an emerging regulator of tissue clearance after stroke (Matlik et al., 2018), and at the cellular level, post-stroke MANF delivery supports many endogenous beneficial cellular repair processes that occur after ischemic stroke (Matlik et al., 2018; Tseng et al., 2018; Gao et al., 2020). Post-stroke MANF administration facilitates neurogenesis (Tseng et al., 2018), promotes innate immunity responses (Matlik et al., 2018), and enhances angiogenesis (Gao et al., 2020). Indeed, all our unbiased approaches with RNA sequencing, metabolomics, and proteomics indicated that MANF mediates innate immunity, and additional studies are needed to clarify the role of both endogenous and exogenous MANF effects. In the rat transient dMCAo model, intracerebral infusion of the recombinant MANF protein starting 3 days post-stroke increased the migration of neural progenitor cells into the infarct region (Tseng et al., 2018). One and 2 weeks after permanent MCAo in rats, Gao et al. found increased angiogenesis and subsequent improvement in cerebral blood flow after recombinant MANF protein administration 1-day post-stroke, possibly due to activation of the vascular endothelial growth factor (VEGF) pathway (Gao et al., 2020). However, we did not find a difference in laminin-positive blood vessel density in the peri-infarct area 2 weeks after transient dMCAo and AAV7-MANF treatment (Matlik et al., 2018). The diverse findings regarding MANF’s angiogenetic properties may be related to differences in the ischemic model used (permanent MCAo vs. transient dMCAo) and the method of MANF delivery (recombinant protein vs. AAV).

To sum up, MANF has both neuroprotective and neurorestorative effects on stroke. The acute mechanisms involve anti-apoptotic effects and, possibly, protection of BBB integrity. The restorative mechanisms may involve neurogenesis, angiogenesis, and immunomodulation. MANF’s effects on immunomodulation and neurogenesis will be further discussed below.

### XBP1 and Modulators of XBP1

Spliced X-box binding protein (XBP1s) is a highly active transcription factor controlling protein translation and UPR; thus it is involved in multiple cellular processes (e.g., maintaining ER homeostasis, immunomodulation, required for autophagy, cell death, and involved in glucose and lipid metabolism) (Hetz et al., 2011, 2020; Walter and Ron, 2011). Xbp1 mRNA expression is induced by ATF6 and spliced by IRE1a in response to ER stress (Yoshida et al., 2001). Xbp1 mRNA is the only splicing target of IRE1a, and removal of 26 nucleotide intron creates functionally active XBP1s (Calfon et al., 2002). The unspliced version of X-box binding protein (XBP1u) is expressed constitutively and acts as a negative feedback regulator during the recovery phase of ER stress; it also maintains cell size (Shaffer et al., 2004). XBP1s upregulates UPR-related genes involved in protein folding, entry to the ER and ERAD, and phospholipid biosynthesis (required for increasing the volume of ER and Golgi). The targets of XBP1s can vary in different tissues or under variable ER stress conditions, as XBP1 can interact and heterodimerize with other transcription factors (Hetz et al., 2011). B-lymphocyte differentiation to plasma cells is dependent on XBP1 (Reimold et al., 2001). Intriguingly, MANF is also widely expressed in secretory cells that require highly developed ER (Lindholm et al., 2008), and MANF is one of the XBP1s target genes (Shaffer et al., 2004; Oh-Hashi et al., 2013; Wang et al., 2018). XBP1 deletion diminishes the mRNA expression of MANF in B-cells (Shaffer et al., 2004). Furthermore, our MANF KO studies have shown a clear link between MANF and XBP1. MANF ablation causes prolonged UPR activation, and sXBP1 is highly upregulated in MANF KO tissues (Pakarinen et al., 2020, 2022). In comparison, neuron-specific MANF expression *in vivo* improved the outcome in the 6-OHDA toxin model of Parkinson’s disease by reducing the levels of p-eIF2a, ATF4, CHOP, XBP1s, GRP78, and ATF6α (Hao et al., 2017). Interestingly, *in vitro*, the positive effect of endogenous MANF overexpression in neurons was achieved by reducing UPR, while the positive effect of extracellularly applied MANF protein was mediated *via* Akt and mTOR (Hao et al., 2017).

The role of XBP1 and UPR in ischemic stroke has been studied for almost 3 decades. As transient ischemic stroke and ER stress cause similar defects, Paschen proposed that the acute detrimental effects of ischemia are caused by disturbed calcium homeostasis causing ER stress and UPR activation in affected cells (Paschen, 1996). Since then, many studies have confirmed the increase in ER stress markers after stroke (Table 2). Initial experiments *in vivo* detected a rapid increase in Xbp1 mRNA and XBP1s levels among other UPR markers after transient forebrain ischemia, indicating disturbance of the ER during damage (Paschen et al., 2003). Following studies with XBP1 transgenic mice confirmed the results and indicated long-term upregulation of XBP1s (signal peaking at 24 h and still present after 3 days). XBP1 expression was detected in glial cells on days 1 and 3 by GFAP co-staining (Morimoto et al., 2007). A similar XBP1 expression pattern was accompanied by the activation of other UPR pathways (Nakka et al., 2010), collectively showing that stroke induces ER stress at the lesion site and penumbra. Clinical relevance was found in genetic studies. The polymorphism of the XBP1 (−116C/G) promoter was investigated in relation to ischemic stroke, atherosclerosis, and hyperhomocysteinemia in human patients, and the XBP1 (−116 G/G) genotype was found to be a risk factor for pediatric ischemic stroke (Yilmaz et al., 2010).

**TABLE 2:** Modulation of XBP1 in stroke.

Model	Treatment	Effect	References
***Clinical studies***			
Pediatric stroke		XBP1 (-116 C/G) gene polymorphism is a risk factor for pediatric ischemic stroke	Yilmaz et al., 2010
***In vivo studies***			
30 or 45 min MCAo, photothrombotic stroke, and 15 min forebrain ischemia in mouse, 60 min MCAo in rat	XBP1 over-expression and knockout, Thiamet-G, glucosamine	Overexpression: UDP-GlcNAc, neurological function ↑ Infarct volume ↓ Knockout: UDP-GlcNAc ↓ Thiamet-G: neurological function in knockout and old and young wt ↑ Glucosamine: neurological function ↑ Infarct volume ↓	Wang et al., 2021b
30 min BCAo in mouse	G-CSF	Neurological and behavioral recovery ↑ p-Akt ↑ ER stress (XBP1 and other markers) ↓ Autophagy, mitochondrial stress, apoptosis ↓	Modi et al., 2020
2 h MCAo in mouse	anti-VEGF antibody	Infarct size, edema, degenerated neurons, apoptosis, IRE1α↓ Neurological function, intact neurons ↑	Feng et al., 2019
30 or 45 min MCAo and pMCAo in mouse	Neuronal XBP1 LoF and GoF, Thiamet-G	LoF: stroke outcome, O-GlcNAcylation ↓ GoF: O-GlcNAcylation ↑ Thiamet-G: O-GlcNAcylation, stroke outcome ↑	Jiang et al., 2017
2 h MCAo in rat	Taurine, DETC-MeSO	GRP78, cleaved ATF6/ATF6 ratio, ATF4, p-IRE1, CHOP ↓ Infarct size, neurological deficit ↓	Prentice et al., 2017a
pMCAo in rat	*Ligusticum chuanxiong, Radix Paeoniae*	Combination: neurological deficit, infarction volume, ER stress (incl. XBP1) ↓ Antioxidant enzyme activity ↑	Gu et al., 2016
2 h MCAo in rat	Taurine, DETC-MeSO	Taurine: p-IRE1, infarct volume ↓ Combination: p-IRE1, infarct volume, neurological deficit, gliosis ↓	Gharibani et al., 2015
2 h MCAo	DETC-MeSO	Cell death, infarction volume, neurological deficit ↓ XBP1 but not p-IRE1 ↓	Mohammad-Gharibani et al., 2014
2 h MCAo in rat	2-deoxyglucose	Neurological function, XBP1 ↑ Infarction volume ↓	Wu et al., 2014
2 h MCAo in rat	rhMANF 20 min pre-stroke	Neuronal loss ↓ Caspase-3 cleavage, apoptosis ↓ BIP/GRP78, p-IRE1, and XBP1 ↓	Yang et al., 2014
2 h MCAo in rat	Taurine	Cleaved ATF6 and its ratio to ATF6, p-IRE1 ↓ Infarct size ↓	Gharibani et al., 2013
2 h MCAo in rat	Salubrinal	Characterization of ER stress gene expression after stroke, including increased Xbp1 mRNA processing Salubrinal: eIF2α phosphorylation ↑ Infarction volume ↓	Nakka et al., 2010
pMCAo in mouse		Characterization of ER stress gene expression after stroke Xbp1 mRNA splicing and XBP expression increased at 6 h to 3 days post-stroke, localized to the neuron cytosol and dendrites, and glial cells	Morimoto et al., 2007
30 min MCAo in mouse		Xbp1 mRNA increased at 1 and 3 h, protein at 6 h in mouse	Paschen et al., 2003
***In vitro studies***			
OGD/R in primary rat microglia and cortical neuron cultures	Icariin, IRE1 overexpression	Microglia: secreted IL-1 β, IL-6, TNF-α↓ Neurons: XBP1u, XBP1s, cleaved caspase-3, p-IRE1α/IRE1α ratio, apoptosis ↓ Cell viability ↑ IRE1 overexpression impaired icariin effect	Mo et al., 2020
LPS-stimulated primary microglia or HAPI cell culture	Microglia: BMSC-co-culture 1-day post-LPS; HAPI: PDGF-AA 1-day post-LPS	Microglia: MANF ↑, reversed if BMSCs are MANF-knockdown HAPI: MANF ↑, miR-30a ↓ XBP1 ↑	Yang et al., 2020
OGD in primary rat brain microvascular endothelial cell culture	DANCR overexpression and knockdown, XBP1 knockdown	DANCR overexpression: proliferation, migration, angiogenesis, XBP1s ↑ Apoptosis ↓ DANCR knockdown: proliferation, migration, angiogenesis, XBP1s ↓ Apoptosis ↑ XBP1 knockdown: proliferation, migration, angiogenesis ↓ Apoptosis ↑ miR-33a-5p directly binds to DANCR and XBP1 and reverses DANCR effects XBP1 overexpression reverses miR-33a-5p effects DANCR/miR-33a-5p/XBP1s activates WNT/β-catenin signaling	Zhang et al., 2020
OGD/R in mouse brain microvascular endothelial bEnd.3 cell culture	VEGF siRNA	Cell viability, proliferation ↑ Apoptosis, ROS, XBP1, GRP78 ↓	Feng et al., 2019
OGD in rat brain microvascular endothelial cell culture	XBP1 overexpression and knockdown	XBP1s and XBP1u upregulated after OGD Overexpression: cell survival, proliferation, migration, angiogenesis, cyclin D1, MMP-2, MMP-9, and PI3K/AKT, ERK, and HIF-1α/VEGF signaling ↑ Apoptosis, cleaved Caspase-3, cleaved Caspase-9 ↓ Knockdown: opposite effects	Shi et al., 2018
Hypoxia/reoxygenation in primary rat cortical neuron and PC12 cell culture	Taurine, sulindac	Taurine: cleaved ATF6/ATF6 ratio, p-IRE1 ↓ Taurine + sulindac: cell viability ↑	Prentice et al., 2017b
Hypoxia/reoxygenation in primary rat neuronal cell culture	DETC-MeSO	Cell viability ↑	Mohammad-Gharibani et al., 2014
Hypoxia/reoxygenation in primary rat cortical neuron culture	Taurine	Cleaved ATF6 and its ratio to ATF6, p-IRE1 ↓ Cell viability ↑	Gharibani et al., 2013
OGD and other *in vitro* ischemia and hypoxia models in rat oligodendrocyte precursor cell line and primary cell culture	XBP1 knockdown	Validation of ischemia models XBP1 knockdown does not affect cell viability in ischemia or hypoxia models	Kraskiewicz and FitzGerald, 2011
Human embryonic kidney and mouse embryonic fibroblast cell culture	XBP1 overexpression and knockout	UPR target genes ERdj4 and p58^IPK^ are XBP1-dependent Identification of other target genes (e.g., MANF)	Lee et al., 2003

*BCAO, bilateral carotid artery occlusion; BIP/GRP78, binding immunoglobulin protein/glucose-regulated protein 78; BMSC, bone marrow mesenchymal stem cells; CHOP, C/EBP homologous protein; DANCR, differentiation antagonizing non-protein coding RNA; DETC-MeSO, S-methyl-N, N-diethyldithiocarbamate sulfoxide; ERK1/2-extracellular signal-regulated protein kinase 1/2; G-CSF, granulocyte colony-stimulating factor; GoF, gain of function; HAPI, highly aggressively proliferating immortalized rat microglia; HIF-1a, hypoxia-inducible factor 1-alpha; IL, interleukin; LoF, loss of function; LPS, lipopolysaccharide; MANF, mesencephalic astrocyte-derived neurotrophic factor; MCAo, middle cerebral artery occlusion; MMP, matrix metallopeptidase; OGD, oxygen-glucose deprivation; p-Akt, phosphorylated protein kinase B; PDGF-AA, platelet-derived growth factor-AA; p-IRE1, phosphorylated IRE1; pMCAo, permanent middle cerebral artery occlusion; rhMANF, recombinant human MANF; ROS, reactive oxygen species; TNF-α, tumor necrosis factor α; UDP-GlcNAc, uridine diphosphate N-acetylglucosamine; VEGF, vascular endothelial growth factor; XBP1, X-box binding protein; wt, wild type; ER, endoplasmic reticulum; OGD/R, oxygen-glucose deprivation and reoxygenation; UPR, unfolded protein response.*

Over the years, different treatments aiming to reduce or modify ER stress during stroke have been tested. For example, a selective inhibitor of eIF2α dephosphorylation, salubrinal, reduced lesion size (Nakka et al., 2010). A week of pretreatment with 2-deoxyglucose improved neurological score within hours and decreased lesion size after 24 h while increasing GRP78 (max at 12 h) and XBP1 protein levels (Wu et al., 2014). Injection of rhMANF 2 h post-stroke reduced the levels of XBP1s, GRP78, and p-IRE1, and enhanced neuron numbers and behavioral recovery while inhibiting caspase 3 (Yang et al., 2014). The protective effect of *Ligusticum chuanxiong, Radix Paeoniae Rubra*, and their combination was tested in rats. A significant decrease in the protein levels of UPR-related factors (XBP1, PERK, ATF6, and CHOP) was detected, accompanied by increased expression of GRP78 and microvessel density (Gu et al., 2016).

Stroke activates O-linked beta-N-acetylglucosamine (O-GlcNAc) modification in the penumbra in neurons of young mice, and XBP1-dependent O-GlcNAc modification was neuroprotective (Jiang et al., 2017). However, the regulation of glucose homeostasis *via* XBP1 is independent of ER stress and acts through XBP1s interaction with FoxO1 (Zhou et al., 2011). Thiamet-G, the activator of O-GlcNAc, improved the outcome in Xbp1 KO mice and induced long-term functional recovery in both young and aged mice. In addition, Xbp1s overexpression by AAVs in neurons induced O-GlcNAcylation, and it improved the outcome after stroke (Wang et al., 2021b).

In adult tissues of mice, XBP1 can regulate the proliferation of endothelial cells and angiogenesis after stroke *via* the VEGF signaling pathway (Zeng et al., 2013). Pretreatment with anti-VEGF antibody reduced edema, infarct volume, and improved neurological scores while decreasing IRE1 pathway and ER stress-mediated apoptosis in mice stroke models (Feng et al., 2019). In contrast, MANF enhanced stroke recovery and angiogenesis by stimulating the VEGF pathway (Gao et al., 2020), depicting quite opposite outcomes while modulating the same pathway.

Taurine is an inhibitory neurotransmitter that can protect from ER-stress in primary cell culture and reduce lesion size in ischemic stroke after 4 days by decreasing GRP78, p-IRE1, CHOP, and caspase-12 levels (Gharibani et al., 2013). Delivery of S-methyl-N, N-diethyldithiocarbamate sulfoxide (DETC-MeSO, partial NMDA antagonist) for 4–8 days reduced cell death and infarct size. After the treatment, a decrease in protein levels of ER stress markers, XBP1, ATF4, JNK, p-PERK, p-eIF2α, GADD34, and CHOP, were detected (Mohammad-Gharibani et al., 2014). Granulocyte colony-stimulating factor (G-CSF) is an anti-apoptotic and immunomodulatory protein that mediates angiogenesis and neurogenesis. Daily dose for 4 days improved the behavior in corner and locomotor tests, and induced the expression of GRP78 while reducing the expression of XBP1, ATF4, ATF6, eIF2α, Caspase 12, and CHOP close to the baseline level after 30 min of bilateral carotid artery occlusion (BCAO) (Modi et al., 2020).

XBP1 mRNA and protein expression is induced by oxygen and glucose deprivation (OGD) in rat oligodendrocyte precursor cells (OPCs) (Kraskiewicz and FitzGerald, 2011), brain microvascular endothelial cells (BMEC) (Shi et al., 2018; Zhang et al., 2020), and primary cortical neurons (Mo et al., 2020) *in vitro.* Surprisingly, XBP1 knockdown had no effect on viability following OGD (Kraskiewicz and FitzGerald, 2011). In a co-culture of microglia and neurons, OGD increased the production of inflammatory cytokines in microglia and apoptosis of primary neurons. Increased mRNA and protein levels of XBP1u and XBPs, as well as phosphorylation of IRE1a, were detected, while icariin, an anti-atherosclerotic drug, reversed the effect by decreasing cytokine production (Mo et al., 2020). In comparison, MANF protects neural stem cells against OGD (Tseng et al., 2018) while reducing the levels of IL-1β, IL-6, TNF-α, and GRP78 in astrocytes (Zhao et al., 2013).

Collectively, the research conducted thus far indicates activation of UPR and cell death pathways after ischemic stroke. In some cases, enhancing XBP1 is beneficial (e.g., 2-deoxyglucose and XBP1 dependent O-GlcNAc); in others, the beneficial effect was accompanied by reduction of XBP1. However, most of the tested compounds affected/modulated cell survival pathways and were not specific for XBP1. Thus, more studies targeting the effect of XBP1s in stroke are needed.

**FIGURE 4:**
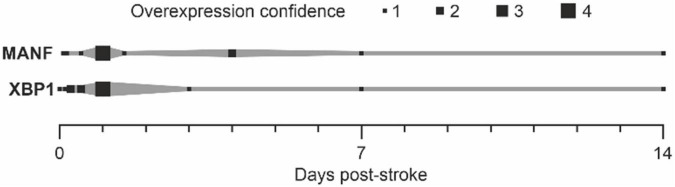
Upregulation of MANF and XBP1 after stroke. “Overexpression confidence” = number of publications showing the upregulation of MANF or XBP1 after stroke (references: Tables 1, 2).

In summary, the expression of MANF and XBP1 after stroke has been detected in several time points (Figure 4). As quantification of the data between publications is difficult, we used the number of publications reporting the expression of MANF or XBP1 at each time point as an indication of gene expression after stroke resulting in an “overexpression confidence” (Figure 4). MANF and XBP1 are both detected right after ischemic stroke, and their expression is still elevated even 2 weeks after the injury.

## Immunomodulatory Effects of MANF

There is very strong evidence that MANF modulates inflammation both *in vitro* and *in vivo* in different disease models and in different model organisms (Table 3). Interestingly, the MANF protein is highly expressed in bone marrow and lymphoid tissues, and *Manf* mRNA is expressed in immune cells including microglia, monocytes, T-cells, B-cells, granulocytes, and dendritic cells to name a few, and the expression is especially high in plasma cells (Karlsson et al., 2021), which could implicate a role in immune cell function. Immune cell activation after ischemic stroke or systemic LPS has been shown to upregulate MANF in microglia (Shen et al., 2012; Sousa et al., 2018).

**TABLE 3:** Immunomodulatory effects of MANF.

Model	MANF therapy	Effect	References
***Ischemia***			
1 h MCAo in aged mice	rhMANF	IL-6, IL-1β, TNF-α↓	Han et al., 2021
OGD on senescent bEnd.3 cells	rhMANF	IL-6, IL-1β, TNF-α↓ TLR4, MyD88, nuclear NF-κB p65 ↓	Han et al., 2021
1 h dMCAo in rat	AAV1-MANF	S100A8; S100A9 ↓	Teppo et al., 2020
2 h MCAo in rat	MANF-knockdown BMSC transplant	M1 markers ↑ M2 markers ↓	Yang et al., 2020
1 h dMCAo in rat	AAV7-MANF	Number of CD68 + and Arg1 + cells ↑ *Emr1*; *C3* ↑	Matlik et al., 2018
OGD in primary rat astrocytes	rhMANF	IL-1β, IL-6, TNF-α↓ GRP78, NF-κB p65 ↓	Zhao et al., 2013
MCAo in rat	–	MANF ↑ in microglia/macrophages	Shen et al., 2012
***Other tissue damage***			
Elective knee arthroplasty in patients	–	Negative correlation between MANF and cytokine levels in the serum after knee operation	Liu et al., 2021
Laparotomy in mouse	rhMANF	IL-1β, IL-6, TNF-α in serum and cortex ↓ Number of Iba1 + and iNOS + cells in cortex ↓	Liu et al., 2021
Light-induced retinal damage in an aging mouse	rhMANF	CD68 + cells in retina ↓	Neves et al., 2020
Alcohol-induced liver injury	–	MANF expression ↑	Chhetri et al., 2020
Liver cancer in humans	–	Colocalization with NF-κB subunit p65 Low MANF levels associated with poor survival	Liu et al., 2020
Old WT mouse	rhMANF or plasmid	Liver inflammation and damage ↓	Sousa-Victor et al., 2019
TBI in rat	rhMANF	IL-1β; TNF-α; NF-κB ↓	Li et al., 2018
Light-induced retinal damage in *D. melanogaster* and mouse	rhMANF	Alternative activation of innate immune cells ↑	Neves et al., 2016
***Inflammation***			
LPS-induced kidney injury in mouse		CD68 + cells in MANF ko ↑	Hou et al., 2021
LPS-induced myocarditis in mouse	rhMANF	Pro-inflammatory markers in myocardium tissue ↓ CD68 + and CCL2 + cells in myocardium tissue ↓	Wang et al., 2021a
LPS-induced C6, MEF, and INS-1E cells	rhMANF	MANF downregulated NF-κB activation via neuroplastin	Yagi et al., 2020
Cytokine-induced damage in human β cells	rhMANF	Apoptosis ↓ NF-κB pathway ↓ UPR ↓	Cunha et al., 2017; Hakonen et al., 2018
LPS-injected mice	–	*Manf* ↑ in microglia	Sousa et al., 2018
TNF-α-induced 293T cells LPS-induced rat primary FLS	plasmid rhMANF –	NF-κB ↓ IL-1β, TNF-α↓ Interaction between MANF and p65 in nucleus	Chen et al., 2015
***Manf removal***			
Monocyte-macrophage-specific MANF KO mouse	LPS-induced kidney injury	CD68 + and CCL2 + cells in kidney ↑ Pro-inflammatory markers in kidney, serum, and peritoneal macrophages ↑ NF-κB activation in kidney ↑	Hou et al., 2021
Monocyte-macrophage-specific MANF KO mouse	LPS-induced myocarditis	Pro-inflammatory markers in serum and myocardium tissue ↑ CD68 + and CCL2 + cells in myocardium tissue ↑ NF-κB activation in myocardium tissue ↑	Wang et al., 2021a
MANF heterozygous mouse	–	CD68 + cells in retinal choroid ↑	Neves et al., 2020
MANF knockdown in C6 cells	–	NF-κB activation ↑	Yagi et al., 2020
Hepatocyte-specific MANF KO mouse	Alcohol-induced liver injury	Liver injury and inflammation ↑ ER stress ↑ Oxidative stress ↑	Chhetri et al., 2020
MANF knockdown in *C. elegans*	–	Changes in innate immunity-related gene expression Altered growth in the presence of bacteria	Hartman et al., 2019
Monocyte-macrophage-specific MANF KO mouse	–	Healthy: Number of M1 macrophages in the spleen ↑ Hepatic fibrosis: Number of M2 macrophages in the spleen ↑	Hou et al., 2019
MANF heterozygous mouse	-	Liver inflammation and damage ↑	Sousa-Victor et al., 2019
Hepatocyte-specific MANF KO mouse	–	Liver IL-1α, TNF-α↑	Liu et al., 2020
MANF knockdown in 293T cells	–	IL-1β, TNF-α↑ NF-κB ↑	Chen et al., 2015
Glial DmMANF knockdown in *D. melanogaster*	–	Appearance of new DmMANF + microglia-like cell type	Stratoulias and Heino, 2015b
MANF-deficient *D. melanogaster*	–	Immune and defense response-related genes ↑	Palgi et al., 2012
***Other***			
*S. domuncula*	–	SDMANF colocalization with Toll-like receptor	Sereno et al., 2017

*AAV, adeno-associated virus; Arg1, arginase 1; C3, complement component 3; CCL2, C-C motif chemokine ligand 2; CD68, cluster of differentiation 68, dMCAo, distal middle cerebral artery occlusion; Emr1, EGF module-containing mucin-like receptor; FLS, fibroblast-like synoviocytes; GRP78, 78-kDa glucose-regulated protein; Iba1, ionized calcium-binding adaptor molecule 1; IL, interleukin; iNOS, inducible nitric oxide synthase; KO, knockout; LPS, lipopolysaccharide; M1, classically activated microglia/macrophages; M2, alternatively activated microglia/macrophages; MAPK, mitogen-activated protein kinase; MCAo, middle cerebral artery occlusion; MEF, mouse embryonic fibroblast; MyD88, myeloid differentiation factor 88; NF-κB, nuclear factor kappa-light-chain-enhancer of activated B cells; NSC, neural stem cell; OGD, oxygen-glucose deprivation; S100A8, calgranulin A; S100A9, calgranulin B; TBI, traumatic brain injury; TLR4, toll-like receptor 4; TNF-α, tumor necrosis factor α; UPR, unfolded protein response; WT, wild type; BMSC, bone marrow mesenchymal stem cells; Dm, Drosophila melanogaster; ER, endoplasmic reticulum; MANF, mesencephalic astrocyte-derived neurotrophic factor; rhMANF, recombinant human MANF; SD, Suberites domuncula.*

The main immunomodulatory effects by MANF recognized so far are: (1) downregulation of pro-inflammatory cytokine production *via* the NF-κB pathway, (2) alteration of phagocytic activity after ischemic stroke, and (3) induction of alternative activation of microglia/macrophages.

Mesencephalic astrocyte-derived neurotrophic factor has been shown to downregulate the NF-κB pathway and decrease pro-inflammatory cytokine production in several *in vitro* studies (Zhao et al., 2013; Chen et al., 2015; Zhu et al., 2016; Cunha et al., 2017; Hakonen et al., 2018; Liu et al., 2020; Han et al., 2021). There is a link between inflammation and ER stress, and activation of the IRE1α UPR pathway, and possibly other UPR pathways, can activate NF-κB (Chaudhari et al., 2014). It is, thus, plausible that endogenous MANF could downregulate NF-κB and downstream cytokine production indirectly by downregulating IRE1α and other UPR pathways in the ER. However, in some studies, MANF was suggested to be the link between ER stress and NF-κB pathway regulation, as direct interaction in the nucleus between endogenous MANF and the transcription factor NF-κB has been implicated (Chen et al., 2015; Liu et al., 2020). MANF is typically localized in the ER lumen, but based on studies with kidney and hepatic cell lines, it was suggested that the membrane protein SUMO1 (small ubiquitin-related modifier 1) would mediate MANF translocation to the nucleus upon ER stress/inflammation, and that the MANF C-terminal domain would inhibit the binding of NF-κB subunit p65 to the DNA (Chen et al., 2015; Liu et al., 2020). Nuclear colocalization of MANF and p65 was also found in liver tissue sections of patients with hepatocellular carcinoma (Liu et al., 2020). MANF was suggested to reduce NF-κB signaling and suppress cancer in hepatocytes (Liu et al., 2020). Yang et al. reported a colocalization of MANF and nuclear marker DAPI in the ischemic brain after transient MCAo in rats (Yang et al., 2020). However, the prevalence of MANF’s nuclear localization is unknown, as it is reported only in few studies. Furthermore, MANF does not have a nuclear targeting sequence, and there is no evidence that exogenous MANF would translocate to the nucleus or that it would be taken up to the ER lumen from the extracellular space. Consequently, MANF has been proposed to bind neuroplastin in the plasma membrane and to block the NF-κB activating effect of neuroplastin (Yagi et al., 2020). It is noteworthy to mention that MANF occupied only about 5–6% of the receptor, and that shRNA for neuroplastin decreased this to 2–3%. This is not very typical receptor binding for proteins with a plasma membrane receptor, e.g., GDNF (Allen et al., 2013).

In an ischemic MCAo stroke model in aged mice of 18–20 months, intracerebroventricular injection of the recombinant MANF protein 2 h after MCAo decreased the production of pro-inflammatory cytokines in the infarct area, reduced neutrophil infiltration into the brain parenchyma, and decreased BBB damage 72 h post-stroke (Han et al., 2021). Using a mouse endothelial cell line, downregulation of the TLR/MyD88/NF-κB signaling pathway was suggested to be behind MANF’s beneficial effect on BBB integrity (Han et al., 2021). In a rat model of traumatic brain injury, MANF treatment decreased the levels of pro-inflammatory cytokines around the contusion (Li et al., 2018). The recombinant MANF protein has been shown to decrease pro-inflammatory cytokines in the liver of old wild-type (WT) mice (Sousa-Victor et al., 2019), and there is evidence from MANF KO mice that the inflammation is increased in the liver upon removal (Chhetri et al., 2020) or downregulation of *Manf* (Sousa-Victor et al., 2019). Removal of MANF from macrophages was shown to increase pro-inflammatory macrophages in the healthy spleen (Hou et al., 2019), and upon LPS treatment in the kidney (Hou et al., 2021) and myocardium tissue (Wang et al., 2021a). Recombinant MANF was able to downregulate inflammation in KO mice after myocarditis (Wang et al., 2021a). Furthermore, intraperitoneal injection of the recombinant MANF protein was shown to downregulate pro-inflammatory cytokines in the serum and prefrontal cortex after abdominal operation in WT mice (Liu et al., 2021). The finding in the brain is interesting, since the brain was not manipulated in any way, and indicates inflammatory crosstalk between the periphery and the CNS, which is a contributing factor in stroke pathogenesis as well. In patients, the same study found implications of a negative correlation between endogenous MANF protein levels and cytokine levels in the serum after knee operation, indicating that low MANF serum levels may contribute to postoperative systemic inflammation (Liu et al., 2021). Another patient study found elevated *Manf* mRNA levels in leukocytes in inflammatory diseases such as rheumatoid arthritis and systemic lupus erythematosus (Chen et al., 2015).

We have shown that Arg1-positive (arginase 1) and phagocytic CD68-positive cells are increased after viral vector-mediated MANF gene delivery in a rat dMCAo cortical ischemic stroke model (Matlik et al., 2018), coinciding with elevated brain repair processes after stroke. Increased number of these innate immune cells was observed in the external capsule and dorsal striatum on post-stroke day 4 after peri-infarct area-targeted MANF gene delivery. The effect was transient, as on day 14 post-stroke there were no differences in the amount of Arg1- or CD68-positive cells in the peri-infarct region. By double immunofluorescence on day 4 post-stroke, the amount of MBP (myelin basic protein) and CD68 double-positive cells was increased in the external capsule of AAV7-MANF-treated rats compared to the AAV7-GFP control group, indicating enhanced phagocytosis of myelin debris after AAV7-MANF treatment. The mRNA levels of innate immunity-related genes *EGF module-containing mucin-like receptor 1 (Emr1)* and *complement component 3 (C3)* were upregulated in the lateral peri-infarct cortex of AAV7-MANF-treated rats at the same time point. However, the mRNA levels of myeloid cell “alternative activation” marker genes *Tgfb1* and *mannose receptor C-type 1 (Mrc1* a.k.a. *CD206)* were unchanged.

Using the same approach of expressing the MANF transgene in the peri-infarct region in the rat dMCAo model (Matlik et al., 2018), we conducted multiomics to study the effects of peri-infarct-targeted AAV1-MANF administration 2 days post-stroke (Teppo et al., 2020). The peri-infarct region was sampled 4 days post-stroke and subjected to untargeted transcriptomics, proteomics, and metabolomics analyses. The most notable effect of MANF was upregulation of transcripts related to immune response, especially toward virus, that could be detected despite the massive inflammation caused by the stroke itself. Interestingly, this effect was not observed with AAV7-MANF, suggesting that it is serotype-specific. The cause for this remains unknown but can be due to the serotypes’ differences in transduction efficiency, time frame, or tissue/cell type specificity. In addition, MANF reversed the stroke-induced upregulation of the innate immunity proteins S100A8 and S100A9, which are highly expressed in phagocytic cells, involved in phagocyte recruitment and released from phagocytes upon activation. By double immunofluorescence, S100A9 was localized to cells resembling neutrophils, but the number of S100A9-positive cells in the peri-infarct region was unchanged between the MANF and eGFP (enhanced green fluorescent protein) control groups. The observed changes can be due to altered RNA and protein dynamics or, more likely, altered proportions of cell populations in the peri-infarct region. Although the mechanisms require further study, both results provide further evidence of the immunomodulatory effects of MANF.

MANF has been found to induce alternative activation of microglia/macrophages, which is considered beneficial in the repair processes of tissue injury. In retinal damage in mice and *D. melanogaster*, the recombinant MANF protein increased the number of alternatively activated pro-regenerative/anti-inflammatory innate immune cells, protected from retinal degeneration, and promoted integration of transplanted photoreceptors (Neves et al., 2016). Importantly, MANF was also shown to modulate inflammation in the retina of aging mice by downregulating the number of CD68-positive cells and similarly protect the retina from degeneration and promote the integration after retinal transplantation in aged mice (Neves et al., 2020). Interestingly, Neves et al. suggested that CX3CR1 is needed for MANF-induced cytoprotection and immunomodulation, as the protective effect was abolished in CX3CR1 KO mouse retina and MANF failed to induce alternative activation in primary macrophage cultures from these mice (Neves et al., 2016). Similarly, in *D. melanogaster*, hemocyte-specific knockdown of the KDEL receptor abolished endogenous MANF’s protective and immunomodulatory effect. Thus, this is another evidence that KDELRs are needed to keep the endogenous MANF in the ER lumen where it acts. Furthermore, MANF was found to be the key regulator in bone marrow mesenchymal stem cell (BMSC)-mediated alternative activation (“M2” polarization) of microglia/macrophages (Yang et al., 2020). In a rat transient MCAo model, treatment with WT BMSCs enhanced behavioral recovery, decreased infarct volume, and increased “M2” polarization, while knockdown of MANF from BMSCs abolished the beneficial effects. Additionally, the BMSC-treated animals had higher number of MANF-positive and Arg1-positive “M2” microglia/macrophages and lower number of iNOS-positive pro-inflammatory “M1” microglia/macrophages than the vehicle-treated or BMSC-MANF knockdown ones 24 h post-MCAo, indicating that the exogenous MANF secreted by BMSCs could act in a paracrine manner and enhance endogenous MANF protein expression in myeloid cells and polarize these immune cells toward the anti-inflammatory “M2” state. However, conflicting results were found in macrophage-specific MANF KO mice with hepatic fibrosis, where the number of alternatively activated spleen macrophages was increased in the MANF KO compared to WT mice (Hou et al., 2019).

Collectively, MANF has immunomodulatory effects, which may be important for the therapeutic function of MANF under different disease conditions. Our results with unbiased methods also clearly demonstrate toward this direction. Mostly, MANF has been shown to downregulate inflammation and removal of *Manf* to increase inflammation in different tissues and cell lines. However, in ischemic stroke, we have reported that MANF induced a transient increase in CD68-positive innate immune cells, which is likely beneficial for tissue repair (Matlik et al., 2018). Therefore, MANF may have a general function aiming to restore tissue homeostasis under pathological conditions. Notably, it has been proposed that MANF affects immune cells in an autocrine/paracrine manner, thus inducing a phenotypic shift toward reparative functions (Neves et al., 2016; Yang et al., 2020). We hypothesize that when MANF is released from injured cells upon ER Ca^2+^ depletion, the released MANF could modulate the immune cell phenotype and, in ischemic stroke, the recruitment of phagocytic cells to the area of injury.

## Neurogenesis

Previously, neurogenesis was thought to occur only during embryonic and perinatal development. However, research findings in the past decade have made it clear that neurogenesis is a process that occurs throughout adulthood, ensuring lost neurons are being replaced by new ones in distinct parts of the brain (Ming and Song, 2011). There are two neurogenic regions in the brain: the subventricular zone (SVZ) and the subgranular layer (SGL) of the hippocampus. In the central nervous system, the SVZ and the SGL regions maintain a distinct population of neural stem cells (NSCs) that show multipotency and higher plasticity. NSCs located in the SVZ give rise to neural progenitor cells (NPCs), giving rise to neuroblasts. From there, these neuroblasts migrate through the rostral migratory system into the olfactory bulb, where they become periglomerular neurons or granules throughout the whole period of adulthood. Newly formed neurons in the process of neurogenesis have diverse and multifaceted functions ensuring olfaction and hippocampal-mediated learning and memory (Aimone et al., 2014). Various factors influence this neurogenesis process, including aging, Alzheimer’s disease, Parkinson’s disease, and stroke. Therapeutic interventions to ensure neurogenesis under different conditions have been a significant area of focus in the field of biomedical research.

In the past, the MANF protein was shown to be predominantly expressed in neuronal lineage cells of the mammalian cortex (Lindholm et al., 2008). Meanwhile, under ischemic or hypoxic conditions, MANF is inducible in glial cells, which are essential for maintaining homeostatic condition and neuronal activity (Shen et al., 2012). Although deletion of *Manf* does not affect the generation of cortical neurons in the developing cortex, it causes a deficit in neurite outgrowth, especially neurofilament-expressed axonal extension (Tseng et al., 2017). In addition, delayed subtype neuron specification and abnormal neuronal density accompanied by 10% reduction of *Manf* KO cortical thickness were found during corticogenesis, suggesting that loss of MANF leads to aberrant neuronal migration and differentiation (Tseng et al., 2017). Showing clinical relevance, the symptoms of a patient with type 2 diabetes mellitus, hypothyroidism, primary hypogonadism, short stature, mild intellectual disability, obesity, deafness, high myopia, microcephaly, and partial alopecia were likely caused by a mutation in MANF (Yavarna et al., 2015). Similar symptoms of deafness, developmental delay, microcephaly, and short stature were found in two patients with childhood-onset diabetes lacking MANF because of mutation in the *Manf* gene (Montaser et al., 2021). In short, endogenous MANF is involved in neuronal migration and neurite outgrowth and may play a crucial role in cortical development. However, it does not seem to affect neurogenesis by modulating the proliferation of neuronal stem cells. To further elucidate the mechanistic action of MANF in neuronal migration, the level of MANF in SVZ explants was manipulated by deleting *Manf*. SVZ cells lacking MANF showed a shorter distance of migration compared with WT cells. Also, we found that administration of MANF could induce neural/glial differentiation accompanied by morphological change in bipolar shape as well as promotion of cell migration in our SVZ explants (Tseng et al., 2018). Furthermore, deletion of *Manf* in NPC cultures was shown to activate UPR signals during neuronal differentiation. These data were among the first to show that UPR can regulate neurogenesis and brain development. This study again suggests a critical role of MANF in ER homeostasis, which is implicated in differentiation of NPCs, development of neurite-like processes, and, subsequently, migration of neuronal cells. It should be noted that the ER extends all the way to the tip of the processes in a neuron (Sree et al., 2021), and that migration of cells and extension of the ER during neuronal development result in enhanced need for ER homeostasis. Although many studies assume that MANF mediates trophic effects in neuronal cells, further studies will be required to address a more mechanistic understanding of MANF’s effect on neurogenesis during brain development.

In previous studies, MANF protein expression was shown to be robustly increased after MCAo (Belayev et al., 2020), and increasing MANF abundance could improve neurobehavioral recovery by means of promoting neuronal survival and modulation of innate immune cells (Matlik et al., 2018). Also, application of the exogenous MANF protein can promote NPC differentiation *in vitro* as well as migration toward the infarct boundary *in vivo* (Tseng et al., 2018). Furthermore, MANF has been shown to have immunomodulatory properties to improve the success of cell-replacement regenerative therapies in a mouse model of degenerative retina (Neves et al., 2016). This neuroregenerative capacity of MANF might be attributed to faster clearance of dead cells and improve the microenvironment. Further studies should be conducted to explore the molecular mechanisms involved in MANF’s regulation of these processes, which may lead to the development of more efficient therapeutic approaches for patients with stroke.

## Future Perspectives

Although studies have proven MANF’s pleiotropic therapeutic effect in several disease models and tissues, the molecular mechanism of action of MANF remains a conundrum. MANF is a neuro-restorative, immunomodulatory protein, and it facilitates behavioral recovery after stroke. As an ER lumen resident protein and a factor needed for maintaining the homeostasis of the ER, the ER is probably the main site of action, but the secretion of MANF and exogenous activity indicate that the mechanism of action is more complicated. The initial hypothesis was that MANF works in the ER to enhance survival/prevent apoptosis. Indeed, the main target of MANF in the ER seems to be UPR modulation *via* the IRE1 pathway in collaboration with other ER proteins (e.g., GRP78 and PDI6), but MANF could also mediate protein transport to/into mitochondria (interaction with CH60) and/or the cytoplasm (KCRB and PGAM). The localization of MANF has been determined based on co-staining with ER resident proteins (e.g., Hrd1, PDI, and GRP78, not always ideally superimposing); thus the interaction of MANF with proteins located in the cytoplasm and mitochondria in other cell types requires further studies, and particularly we should show localization of endogenous MANF by electron microscopy.

MANF interacts with GRP78, PDI6, and ATP, all modulating UPR activation in a specific manner; thus, MANF functions as a negative feedback regulator fine-tuning ER homeostasis. However, in the brain, *Manf* mRNA is expressed in many cells at high level, but the MANF protein is expressed only in neurons. This is reversed in the case of an injury, indicating cellular differences that may be originating from the complexity of the ER and may provide a protective mechanism in case of a disturbance at tissue level. It should be studied further, are high mRNA levels of *Manf* present in a cell as a resource to translate it promptly when needed? Also, it is not known why or how the elevated levels of *Manf* mRNA are maintained in glial cells. In addition, we should investigate whether the phenomenon observed in our model systems also exists in human cells, as even for conserved proteins this is not always the case. Since the mechanism of action for MANF seems to be broad, we should be careful and accurate when discussing ER luminal effects or extracellular effects of MANF. As it is well-known that proteins can interact with many partners after overexpression, it is important to perform studies at physiologically relevant concentrations when we investigate and determine the mechanism of action of MANF. In the ER lumen, MANF has been shown to act as an ER chaperone, and other mechanisms of action have been postulated. We should further clarify what the primary functions of the native protein are, e.g., is it so that the majority of proteins is fulfilling some function in homeostasis and the minority is doing something else, and how the function/action changes during increased folding load demand in the ER lumen. Furthermore, when clarifying the mechanisms of action, we should implement pharmacological inhibitors, activators, siRNA and overexpression, and CRISPR deletion experiments to show robustly that the downstream effect is mediated *via* proposed way. Instead of conducting more correlative studies, it would be more beneficial to actually show the mechanism of action with tools that are already available.

Comparative studies on MANF in combination with XBP1 would shed more light on whether and how the pathways are connected and what the mechanism is, and how the modulation of UPR and inflammatory pathways improves recovery. Also, more studies would be needed where the IRE1 pathway is inhibited, deleted, increased, or overexpressed together with MANF administration to reveal whether XBP1 is mediating MANF’s beneficial effects. As stroke is more prevalent in the elderly and aging causes reduced protein folding capacity, it would be of interest to discover how the regulation of XBP1 and MANF (and/or interactions) remains the same in aged animals. Innovative technologies for single-cell analysis are emerging, and they could provide more information on functional similarities and differences in cellular level *in vivo*. Alternatively, isolation of the nucleus and snRNA-seq could be conducted. Cell isolation and separation are lengthy processes, thus, one option would be to use primary cell cultures (e.g., microglia, astrocytes) or iPSC of neurons (as isolation of intact neurons is difficult) to study the underlying molecular mechanisms. Still, all new genomic tools will enhance data load but may not be specific enough to detect genes of interest. We would need to develop specific antibodies for all UPR arm proteins that could be used to study how UPR is regulated in a time-dependent manner after stroke in the peri-infarct area and in which cells. Furthermore, it would be important to clarify which UPR arms are activated in dendrites, axons, and presynaptic and postsynaptic terminals after ischemic injury. Lastly, we should determine the role of UPR in a cell-specific manner and take into account whether chronic increase in ER folding quantity or quality can lead to degeneration of a cell.

## Author Contributions

HL and MA conceived the review. HL, JA, H-KL, K-YT, JT, VS, and MA wrote the manuscript. All authors revised and approved the final version of the manuscript for submission.

## Conflict of Interest

The authors declare that the research was conducted in the absence of any commercial or financial relationships that could be construed as a potential conflict of interest.

## Publisher’s Note

All claims expressed in this article are solely those of the authors and do not necessarily represent those of their affiliated organizations, or those of the publisher, the editors and the reviewers. Any product that may be evaluated in this article, or claim that may be made by its manufacturer, is not guaranteed or endorsed by the publisher.
